# ^3^*He* Spin Filter for Neutrons

**DOI:** 10.6028/jres.110.042

**Published:** 2005-06-01

**Authors:** M. Batz, S. Baeßler, W. Heil, E. W. Otten, D. Rudersdorf, J. Schmiedeskamp, Y. Sobolev, M. Wolf

**Affiliations:** Institut für Physik der Universität Mainz, D-55099 Mainz, Germany

**Keywords:** ^3^He polarizer and compressor, neutron spin filter, optical pumping, relaxation, parity violation, polarized neutrons, polarization analysis

## Abstract

The strongly spin-dependent absorption of neutrons in nuclear spin-polarized ^3^He opens up the possibility of polarizing neutrons from reactors and spallation sources over the full kinematical range of cold, thermal and hot neutrons. This paper gives a report on the neutron spin filter (NSF) development program at Mainz. The polarization technique is based on direct optical pumping of metastable ^3^He atoms combined with a polarization preserving mechanical compression of the gas up to a pressure of several bar, necessary to run a NSF. The concept of a remote type of operation using detachable NSF cells is presented which requires long nuclear spin relaxation times of order 100 hours. A short survey of their use under experimental conditions, e.g. large solid-angle polarization analysis, is given. In neutron particle physics NSFs are used in precision measurements to test fundamental symmetry concepts.

## 1. Introduction

Polarized neutron scattering plays a key role in the microscopic understanding of the static and dynamic properties of magnetic materials. The main limitations which prevent so far a broad application of neutron polarization analysis studies are the low counting rates involved and the severe restrictions regarding the range of energy transfer and scattering angle available; in other words the phase space which can be covered by existing polarizer and analyzer devices. Polarizing filters using gaseous nuclear spin-polarized ^3^He operate by selectively removing one of the neutron spin states of an incident beam and allowing the other spin state to be transmitted with only moderate attenuation and are thus a promising polarizing/analyzing tool for neutrons over the full spectrum of cold, thermal and hot neutrons.

The outline of this paper is organized in the following way: Sec. 2 describes the principle of a ^3^He neutron spin filter (NSF) and gives basic information. In Sec. 3 the Mainz ^3^He polarizer and compressor and its performance is presented. Two examples of NSF applications are discussed in Sec. 4 followed by the conclusion (Sec. 5).

## 2. Principle of a ^3^He Neutron Spin Filter

The principle underlying the operation of polarized ^3^He filters as neutron polarization filters is the large nuclear spin-dependence of the neutron-capture into a broad (*Γ* = 270 keV) unbound resonance (*J^π^* = 0^+^) in the intermediate ^4^He* state (^3^He + *n* → ^4^He*) which decays to *T* + *p* with an energy release of 740 keV. Only neutrons with spin component antiparallel to the ^3^He nuclear spin for which the capture cross section is very high (*σ* ↑↓ [barn] ≈ 6000 · λ [Å]) are absorbed in this reaction. Neglecting the small potential scattering of the neutrons with spin component parallel to the ^3^He nuclear spin (σ ↑↑ [barn] ≈ 5), the transmission *T*_±_ for both spin orientations through a cell with polarized ^3^He is given by

$$T_{\pm }=\exp\{-(1\mp P_{\text{He}})\cdot n_{\text{He}}\cdot \sigma_{0}\cdot l\}$$ (1)

where *P*_He_ is the ^3^He nuclear polarization, *n*_He_ the number density of ^3^He atoms, *σ*_0_ the absorption cross section for unpolarized neutrons (*σ*_0_ [barn] ≈ 3000 · λ [Å]) and *l* is the length of the spin filter cell. With + (−) we define the neutron spin component parallel (antiparallel) to the ^3^He spin.

With *T*_±_ one can define three other characteristic parameters which describe the performance of a ^3^He polarizing (analyzing) NSF: the transmission *T_n_* for an unpolarized beam which is given by

$$T_{n}=\frac{T_{+}+T_{-}}{2}=\exp(-O)\;\cdot \cosh(O\cdot P_{\text{He}})$$ (2)

where *O* = *n*_He_ · *l* · *σ*_0_ is the opacity of the filter. For gaseous ^3^He, *O* may be expressed in convenient units by *O* = 0.0732 · *O*’ with *O*’ = *p*_He_ [bar] · *λ* [Å] · *l* [cm] (*p*_He_ : ^3^He gas pressure at 20 °C and *λ* : neutron wavelength). The filter opacity *O*’ is a very useful parameter showing one of the advantages of NSFs because unlike supermirrors, NSFs provide their performance over the full kinematical range of cold, thermal and hot neutrons, since for any neutron wavelength *λ* the filter opacity *O*’ can be set to its optimum value simply by adjusting the product of the ^3^He pressure *p*_He_ and the length of the cell *l*.

The second characteristic parameter is the polarizing (analyzing) efficiency *P_n_* and thus also the resulting neutron polarization which can be expressed by

$$P_{n}=\frac{T_{+}-T_{-}}{T_{+}+T_{-}}=\tanh(O\cdot P_{\text{He}})$$ (3)

The third characteristic parameter is the figure of merit *Q* which is given by

$$Q=T_{n}\cdot P_{n}^{2}$$ (4)

**Fig. 1:**
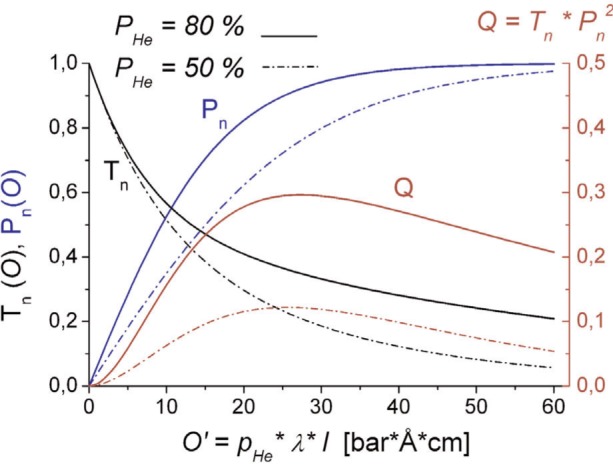
Performance of a ^3^He NSF: total transmission (*T_n_*), polarizing (analyzing) efficiency (*P_n_*) and figure of merit (*Q*) as a function of the filter opacity *O*’. Solid lines: characteristic parameters for a ^3^He nuclear spin polarization of *P*_He_ = 80 %; dashed-dotted lines: *P*_He_ = 50 %.

Figure 1 shows the NSF characteristics as a function of the filter opacity *O*’ for different values of the ^3^He nuclear polarization *P*_He_ (solid lines: *P*_He_ = 80 %; dashed-dotted lines: *P*_He_ = 50 %). The optimum of the filter opacity *O*’ is ≈ 26 bar · Å · cm. For further details, see Refs. [1], [2] and [4].

## 3. Mainz ^3^He Polarizer and Compressor

Our NSF-concept includes a remote type of operation where the ^3^He is spin-polarized in a central production facility from where it is transported to the neutron beam facility for example in specially designed *µ*-metal transport boxes with a homogeneous holding field. After usage the gas can be recovered, the cells can be refilled with freshly polarized gas and the cycle starts again. For this remote type of operation, long *T*_1_ relaxation times (of order 100 hours) of the NSF cells are mandatory and can be achieved with cesium-coated quartz cells for example. One advantage of the concept is that the NSF cells can be used at different instruments (which requires high production rates) and furthermore, the concept allows a high flexibility in NSF cell design.

**Fig. 2:**
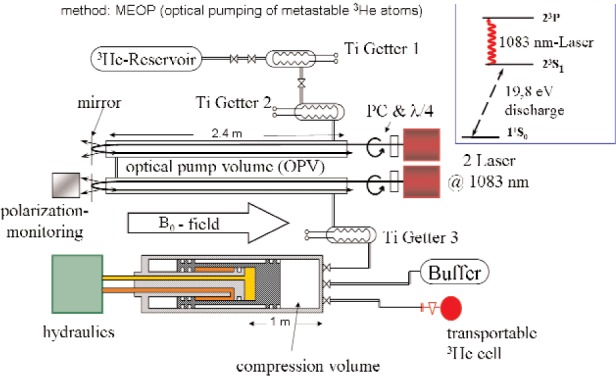
Sketch of the Mainz ^3^He Polarizer and Compressor. The whole apparatus is located in a homogenous magnetic field of 8 G. In reality, we have five optical pumping cells with a total volume of 36 L. For further explanations, see text.

Figure 2 shows a schematic sketch of the Mainz ^3^He polarizer and compressor. We are polarizing the ^3^He gas by Metastability Exchange Optical Pumping (MEOP) where the atoms from the ground state are excited into the metastable 2^3^*S*_1_ state by a discharge and can then be pumped by a 1083 nm-Laser.

The whole apparatus is located in a homogeneous magnetic field of 8 Gauss (relative field gradients (d*B_r_*/d*r*)/*B*_0_ in the order of 10^−4^ cm^−1^), which serves as a holding field and thus as quantization axis for the polarized ^3^He-nuclei.

The polarizing and compressing system consists of three parts:

- The first part (upper part in Fig. 2) contains the ^3^He-reservoir and titanium getters for purification.
- The second part (middle part in Fig. 2) consists of the optical pumping volume with typical pressure values of about 1 mbar. The optical pumping itself is done by two commercial 15 W fibre lasers (IPG Photonics Corporation, Model: YLD-15-1083) at 1083 nm. After having passed a polarizer cube and a lambda-quarter-plate, the laser light is circularly polarized and is then absorbed by the metastable atoms; in this absorption-process, the angular momentum of the photon is transferred to the electron shell of the atom. After reemission and hyperfine-coupling (in the 2 ^3^S_1_ state), the resulting nuclear polarization is transferred to the ground state by metastability exchange collisions. In order to maximize the light absorption, the five OP-cells have a length of 2.40 m and the laser light is back-reflected at dichroic mirrors so that it passes through each OP-cell for a second time. The nuclear polarization of the ^3^He gas can be monitored during the whole OP-process by measuring the circular polarization of the 668 nm-light emitted by the discharge.
- The third part (lower part in Fig. 2) contains a mechanical polarization-conserving compressor driven by hydraulics in order to achieve gas pressures up to 5–6 bar. In a first step, the polarized gas is compressed into a buffer cell of *V* = 4 L. After having polarized the desired amount of gas, the polarized ^3^He from the buffer cell is then compressed in a second step into a detachable storage cell, e.g. a NSF cell.

**Fig. 3:**
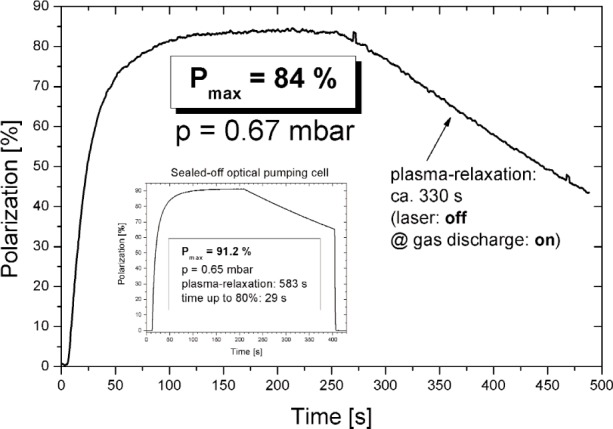
Build-up of ^3^He nuclear polarization in the OP-volume of the polarizer. Inset: Build-up of ^3^He nuclear polarization in a sealed-off OP-cell. The maximum polarization of 91,2 % is higher than in the open system of the polarizer due to gas purity reasons.

**Fig. 4:**
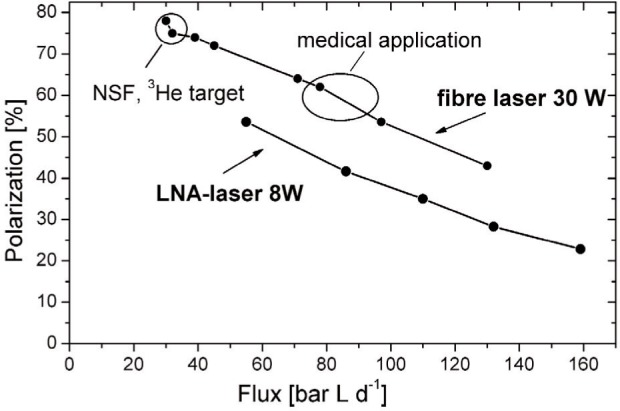
Performance of the Mainz ^3^He polarizer and compressor with the old (LNA-laser 8W, lower line) and the new (fibre-laser 30 W, upper line) laser system. The nuclear polarization is plotted versus the flux (in bar L d^−1^).

Figures 3 and 4 show the performance of the polarizer. The build-up of the ^3^He nuclear polarization as a function of time is shown in Fig. 3. A maximum value for the steady state polarization of (84 ± 2) % (mainly non-statistical uncertainties) was achieved in the fifth OP-cell at a pressure of 0.67 mbar. The relaxation time inside the plasma (with the laser turned off and the gas discharge turned on) was 330 s. In a sealed-off OP-cell at comparable conditions we achieved a maximum polarization of (91 ± 2) % which is higher than in the open polarizer system due to gas purity reasons. In Fig. 4, the polarization measured in the OP-volume is plotted as a function of the flux. The upper line shows the results for the already mentioned 30 W-fibre laser-system, the lower line is for an old 8 W-LNA laser-system. At a flux of 30 bar L/d, the polarization reaches 75 % to 80 %; this is the regime we have to work in for fundamental physics applications like the ^3^He NSF. For the medical application, the MRI of the lungs, where higher production rates are required and lower polarization values around 60 % are sufficient, we work in a regime of 80 to 100 bar L/d.

**Fig. 5:**
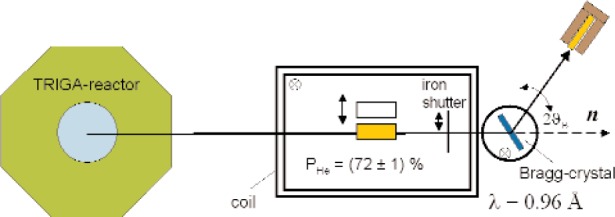
Experimental setup for NSF-tests at the Mainz TRIGA-reactor. The polarized ^3^He cell (lower filled cell) is placed in the thermal neutron beam in a magnetic holding field. The *n*-beam undergoes then a Bragg-reflexion and is monitored by a detector. From the measurement of the *n*-transmission *T_n_* through the ^3^He NSF cell, the ^3^He nuclear polarization was determined: *P*_He_ = (72 ± 1) %. The upper empty cell indicates that the measurement also has to be performed with an evacuated NSF cell in order to determine the neutron transmission of the quartz glass.

Finally, we would like to report on recent NSF-tests performed at the TRIGA-reactor in Mainz in this section. For these NSF-tests, a cylindrical cesium-coated quartz cell with a *T*_1_ relaxation time of 165 hours was used. The ^3^He gas was polarized up to (75 ± 2) % of nuclear polarization measured in the OP-cells. Afterwards the NSF cell was filled with 1.6 bar ^3^He without significant losses and then transported to the TRIGA-reactor where it was put into the thermal neutron beam. The experimental setup is shown in Fig. 5. From the measurement of the neutron-transmission *T_n_* through the NSF cell, one can determine the ^3^He nuclear polarization from Eq. (2) if the ^3^He pressure *p*_He_, the neutron wavelength *λ* and the length of the cell *l* are known. The product of these three parameters, the so called filter opacity *O*’, can be determined experimentally by measuring the transmission *T_n_* of the depolarized cell (*P*_He_ = 0 %) [3]. The ^3^He nuclear polarization measured was *P*_He_ = (72 ± 1) % (mainly statistical uncertainties). This value was checked and confirmed by a second method, which consists in measuring the neutron polarization *P_n_* via Bragg scattering on a CoFe crystal with known analyzing power. By use of Eq. (3) it is possible to determine the ^3^He nuclear polarization. With this value for *P*_He_, we have reached the design-specifications of ^3^He neutron spin filters (cf. Fig. 1) and demonstrated that we are able to use NSF-cells in a remote type of operation with a ^3^He polarization of more than 70 %.

## 4. Two Examples of NSF Applications

### 4.1 Large Solid-Angle Polarization Analysis

**Fig. 6:**
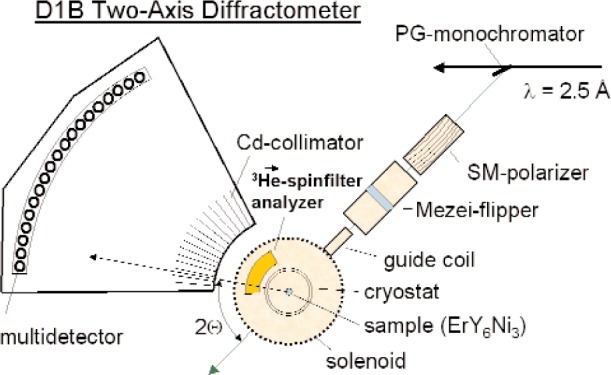
Large solid-angle polarization analysis: Experimental setup at the D1B-two axis diffractometer at ILL. Neutrons with a wavelength of 2.5 Å are polarized by a supermirror, pass a spin flipper and are then scattered at a ErY_6_Ni_3_-sample. Afterwards, they pass the ^3^He NSF analyzer and are monitored by a multidetector.

**Fig. 7:**
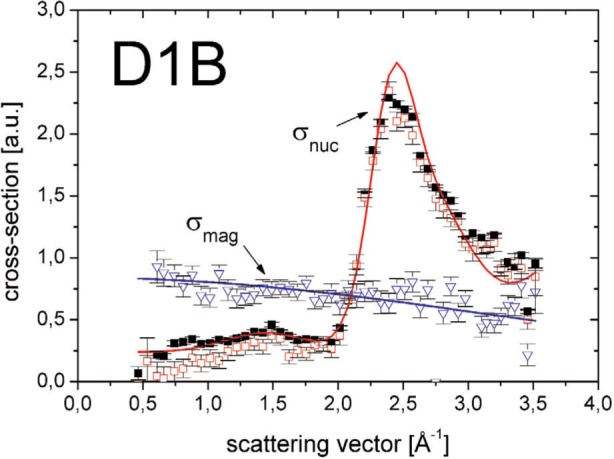
Extracted nuclear and magnetic cross-sections as a function of the scattering vector *Q*. For details, see text and [4].

The principle idea of large solid-angle polarization analysis is the following: an incident polarized neutron beam is scattered at a sample. Very close to the sample, a ^3^He NSF is used as an analyzer which covers the total angular acceptance of the instrument. First measurements using a banana shaped ^3^He NSF cell were performed at the D1B-two axis diffractometer at the ILL in Grenoble [4]. Figure 6 shows the experimental setup, where the polarization of the incident beam was periodically flipped measuring the spin-dependent and the non-spin-dependent cross-sections. From these cross-sections, both the nuclear and the magnetic scattering cross-sections could be extracted. Without going into the details of data taking and data analysis which are described in Ref. [4], one can conclude that the ^3^He NSF has succeeded in providing a clean separation of the magnetic and nuclear scattering cross-sections (see Fig. 7). The results are comparable with those of a corresponding measurement at the D7-diffuse scattering spectrometer at ILL which uses supermirror bender analyzers. For a detailed quantitative comparison of D1B and D7 results see Ref. [4]; for a more general comparison study between spin filters and supermirrors see Ref. [5].

### 4.2 Parity Violating (PV) Spin Rotation and Dichroism in ^139^La and Light Nuclei

The technique of neutron spin analysis based on a ^3^He NSF has also been successfully applied to measure the PV spin rotation and dichroism in ^139^Lanthanum near the *p*-wave resonance at 0.75 eV [6]. The dichroism was determined by measuring the neutron-absorption in the La-target for the two different spin states. The principle to measure the spin rotation was the scheme of crossed polarizer and analyzer widely used in classical optics. The results obtained are consistent with theory predictions. For details of the experimental setup and a discussion of the results see Ref. [6].

**Fig. 8:**
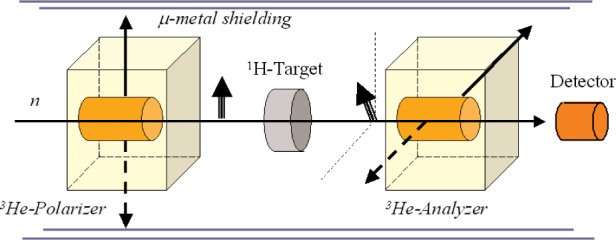
^3^He NSF as neutron spin-flipper for high precision PV-experiments on light nuclei. The neutron spin can be flipped via an adiabatic fast passage (AFP)-spin flip of the ^3^He nuclei.

The PV-effects in La, which is a complex system, are relatively large due to the resonance enhancement near the peak of the *p*-wave resonance. In order to learn something about parity violation and weak interaction, it would be very interesting to study PV on light nuclei like ^4^He or hydrogen, where the weak matrix element can be calculated. The PV-effects, however, are several orders of magnitude smaller. For such high precision PV-experiments on light nuclei, a possible development of the experimental technique is the use of two polarized ^3^He NSF cells, one as polarizer, the second one as analyzer, for example in an experiment to measure the PV-spin rotation. The principle idea is sketched in Fig. 8. The advantage is that one could use the ^3^He NSF as neutron spin flipper, too, because it is possible to flip the spin of the ^3^He nuclei by means of adiabatic fast passage (AFP) and thus the spin of the neutrons. This would reduce significantly the main source of systematic errors, i.e. correlated effects due to the flipping of the *n*-spin by means of classical neutron spin flippers where the magnetic field configuration may vary depending on the respective spin state. The signal losses in the ^3^He polarization caused by AFP can be made very small and were measured to be (0.03 ± 0.01) % per AFP spin-flip [7].

## 5. Conclusion

A ^3^He NSF is a powerful tool for neutron polarization and polarization analysis over the full kinematical range of cold, thermal and hot neutrons. Two examples, the large solid-angle polarization analysis and the use of a ^3^He NSF as neutron spin flipper for high precision PV-experiments are given.

Our concept includes a remote type of operation where the ^3^He is polarized in a central production facility by MEOP and compressed afterwards in detachable NSF cells. The Mainz polarizer reaches a production rate of 30 bar L d^−1^ for high polarization values between 70 % and 80 % (measured in the OP-volume). For this type of operation, long *T*_1_ relaxation times (> 100 h) are mandatory and can be achieved for example with cesium-coated quartz cells which allow a high flexibility in cell design and which have a high neutron transmission.

We reported on recent NSF-tests at the TRIGA-reactor in Mainz which demonstrated that we achieve standard ^3^He nuclear polarization values of more than 70 % regularly after the transport of the NSF-cells from the central production facility to the experiment. These high ^3^He polarization values open up the possibility to widely use ^3^He spin filters for neutron polarization and polarization analysis in particular in the thermal energy range.
